# Psychological Abuse and Social Support in Chinese Adolescents: The Mediating Effect of Self-Esteem

**DOI:** 10.3389/fpsyg.2022.852256

**Published:** 2022-03-24

**Authors:** Chen Chen, Shengkai Ji, Juan Jiang

**Affiliations:** ^1^Center for Educational Science and Technology, Beijing Normal University at Zhuhai, Zhuhai, China; ^2^Teachers’ College, Jiaxing University, Jiaxing, China; ^3^Department of Preschool Education, Liaoning National Normal College, Shenyang, China

**Keywords:** psychological abuse, self-esteem, social support, mediating effect, adolescents

## Abstract

Although previous studies have explored relationships between psychological abuse and social support, the pathways from psychological abuse to social support are still unclear, particularly in Chinese adolescents. This cross-sectional study attempts to delineate the prevalence of psychological abuse and explore the relationships between psychological abuse, social support, and self-esteem under the Chinese cultural context. Data were obtained from 417 Chinese adolescents aged 15–18 years old. All of them completed the Child Psychological Abuse and Neglect Scale, Rosenberg Self-Esteem Scale (SES), and Multidimensional Scale of Perceived Social Support (MSPSS). Results indicated that the prevalence of psychological abuse in Chinese adolescents was 25.66%, and psychological abuse was negatively associated with self-esteem and social support, respectively. Self-esteem partially mediated the relationships between psychological abuse and social support. Findings highlight the importance of improving self-esteem in survivors of psychological abuse for decreasing the negative effects on social support. Additionally, the significance and limitations of the results were discussed.

## Introduction

Child abuse and neglect of all kinds have been an important worldwide public health issue for its high prevalence and negative outcomes (e.g., World Health Organization [WHO], 2014; Cui and Liu, 2018; Christ et al., 2019). Psychological abuse, a subtype of child abuse and neglect, reflects the biased interactions between children and their caregivers, such as scolding, disparage, and threatening. About 36% of children are psychologically abused each year (World Health Organization, 2017), which may contribute it being a common subtype of child abuse and neglect across nations and cultures. Although the relationships between physical and/or sexual abuse and later development have been explored by some studies (e.g., Chen and Qin, 2020a), there is only few knowledge about the relationships and pathways between psychological abuse and later development.

Social support is a crucial factor for coping with stress in daily life, and high social support is a protective factor for later development. Some previous studies have explored the relationships between psychological abuse and social support (e.g., Gu et al., 2014; Sheikh et al., 2016), but there is little knowledge about the pathways of these two variables. Moreover, the prevalence of psychological abuse in Chinese adolescents is inconsistent. Attachment Theory, a theory for parent-child relationships, posits that biased attachment relationships are related with disorganized internal work models that may later influence interpersonal relationships (Ainsworth, 1979). Psychological abuse may reflect the disorganized parent-child relationships and impair later interpersonal relationships. Therefore, guided by Attachment Theory, we attempt to delineate the prevalence of psychological abuse and explore pathways from psychological abuse to social support in Chinese adolescents.

### Prevalence of Psychological Abuse

Psychological abuse, one subtype of child abuse and neglect, has been explored for several decades. Its prevalence has been an important topic for studies (Meinck et al., 2016; Prino et al., 2018; Kumar et al., 2019). Some studies reported that about one in 10 children was psychologically abused in high-income countries (Gilbert et al., 2009), with higher rates in the Pacific region and Asia than in high-income counties (United Nations Children’s Fund, 2012).

China, as an ancient Eastern country, has a different culture from Western countries. This difference may contribute to different prevalence of psychological abuse. Some previous Chinese studies delineated the prevalence of psychological abuse. Hence, the results were inconsistent (ranging from 16.5 to 80.7%; Wang et al., 2012; Gao et al., 2017; Sun et al., 2019). Most studies used data from adults with retrospective questionnaires, which may have caused an overestimation of the prevalence. Chinese parentings may be influenced by the Chinese traditional culture which may contribute to their preference for parents’ punitive discipline for children (Wang et al., 2018). Moreover, the Chinese traditional culture emphasizes the strict hierarchy in the family, which may influence the interactions between children and caregivers and, in turn, increase the risk of psychological abuse.

Meanwhile, the prevalence of psychological abuse may be different between genders. Some studies indicated that girls were more likely to be psychologically abused than boys (Wang et al., 2012; Feng et al., 2015), while others reported the opposite (Choo et al., 2011; Häuser et al., 2011; Charak and Koot, 2014). Boys and girls have different social roles based on the Chinese traditional culture, which may cause different parenting styles between genders. Boys may be persuaded to suppress emotions in daily life, contributing to few interactions between parents and sons. Thus, we hypothesize that prevalence of psychological abuse in China is higher than Western countries, and men may be more likely to get high scores on psychological abuse than women do under the Chinese cultural context.

### The Relationships Between Psychological Abuse and Social Support

Social support can be defined as a social network based on an individual’s perceptions or experiences, which may provide effective emotional and substantial supports when needed (Shi et al., 2017). Social support may be influenced by individuals’ experiences and behaviors (Schwartz et al., 2010). Particularly, adverse childhood experiences (Sperry and Widom, 2013; Rodriguez and Tucker, 2015). Psychological abuse, as one kind of adverse childhood experience, reflects poor or biased interactions between children and their caregivers that may contribute to poor interpersonal relationships which may lead to low social support.

Moreover, individuals who were psychologically abused by their caregivers may have biased internal work models about interactions with the same caregivers, which may extend these perceptions into other relations. These disorganized interpersonal relationships may therefore impair their social support. Some previous studies have explored the relationships between psychological abuse and social support. These studies suggested that individuals with psychological abuse had low levels of social support (Tian, 2014; Goodman et al., 2016; Jin and Wang, 2017; Zhang et al., 2017). For example, Struck et al. (2020), based 580 young adults, indicated that psychological abuse was negatively associated with social support. Similarly, Zhang et al. (2018), based on 909 Chinese college students, reported that psychological abuse was negatively associated with social support.

Although a growing body of studies has explored the relationships between psychological abuse and social support, few studies have explored the pathways between the two, particularly in Chinese adolescents. Influenced by the Chinese traditional culture, Chinese may prefer regarding interpersonal relationships as an important component in daily life. In addition, the quality of interpersonal relationships may represent levels of social support. Hence, the influence of psychological abuse on social support may be severe in Chinese people. We hypothesize, therefore, that psychological abuse impairs individuals’ social support under the Chinese culture.

### Self-Esteem as a Mediator

Self-esteem is a basic inner strength of self-awareness. It is the concept of overall self-worth and signifies a positive or negative comprehensive evaluation of oneself (Cast and Burke, 2002). The development of self-esteem may be influenced by a lot of factors. Particularly, interpersonal relationships may be an important factor for it. Psychological abuse, as chronic stress in daily life, may reflect the biased parent-child relationships. Hence, these poor parent-child relationships may impair the development of self-esteem (Wang et al., 2002). Some previous studies have explored the relationships between psychological abuse and self-esteem. For instance, some studies indicated that psychological abuse impaired the development of self-esteem (Malik and Kaiser, 2016; Waldron et al., 2018), while others did not confirm these results (Bolger et al., 1998).

Moreover, the levels of self-esteem may influence the levels of social support, thereby impairing the individuals’ resilience for facing adversities. Previous studies have explored the relationships between self-esteem and social support (Yildiz and Karadaş, 2017; Ji et al., 2019). For example, Martínez-Martí and Ruch (2017), based on 363 adults in a cross-sectional study, found that self-esteem was positively associated with social support. Li et al. (2018) also reported that self-esteem was associated with social support based on 262 college students.

Additionally, studies have suggested and explored how self-esteem is a mediator in the relationships between child abuse and later development (e.g., Chen and Qin, 2020b). Survivors of psychological abuse may have low levels of self-esteem (Waldron et al., 2018), causing them to be estranged from others and have biased valuations about themselves or interpersonal relationships. This, in turn, influences their social supports (Martínez-Martí and Ruch, 2017; Li et al., 2018). Therefore, we propose that self-esteem may play as a mediator in the relationships between psychological abuse and social support under the Chinese culture context.

### The Present Study

Although psychological abuse has been explored in recent years, few studies have explored psychological abuse among Chinese samples. Therefore, we aim to (1) delineate the prevalence of psychological abuse, (2) verify the relationships between psychological abuse and social support, and (3) explore the role of self-esteem in the relationships between psychological abuse and social support. Thus, we hypothesize that (1) the prevalence of psychological abuse in Chinese adolescents is higher than in Western countries, and that Chinese boys are more likely to get high scores on psychological abuse; (2) psychological abuse is negatively associated with social support; and (3) self-esteem mediates the relationships between psychological abuse and social support.

## Materials and Methods

### Participants and Procedures

We recruited 450 students from two high schools in Jiangsu Province, China to attend the current study. We removed 33 questionnaires with more than 15% missing data. After this exclusion, a final total of 417 participants were included in the current study. Of those participants, 48.20% of them were girls (*n* = 201). The mean age was 16.76 years (SD = 0.83), with a range of 15–18 years. Twenty-six-point-fourteen percent of them (*n* = 109) were an only child in the family. Regarding family income, 11.99% of participants were from families with monthly income below ¥1,000 ($149.07), with 39.09% falling between ¥1,001 ($149.21) and ¥3,000 ($447.21), 31.65% falling between ¥3,001 ($447.36) and ¥5,000 ($745.35), and 17.27% falling above ¥5,000.

The study was approved by the ethics committee of the authors’ institution. The participants, teachers, and caregivers were informed of the research purposes and all participants were asked to sign an informed consent before data collection. The consent form was used to inform participants about the purposes of the study, the confidentiality of their responses, and the use of their data for research purposes. The authors asked the teachers to send the questionnaires to the participants, and the participants completed the questionnaires in their classrooms within 20 min, and a small gift ($.75) was given as compensation.

### Measurements

#### Child Psychological Abuse and Neglect Scale

The Child Psychological Abuse and Neglect Scale (CPANS), developed by Deng et al. (2007), is a 31-item self-reported scale which is used to collect data on psychological abuse and neglect. The CPANS consists of two subscales: 14-item psychological abuse subscale with three dimensions—scolding, threatening, and intervening; and a 17-item psychological neglect subscale with three dimensions—emotion neglect, education neglect, and supervision neglect. Participants were to rate each item (e.g., “*My parents call my names when I did not expect it*,” “*My parents didn’t answer my questions*”) using a 5-point scale (from 0 = *very conformity* to 4 = *very inconformity*) and ratings were averaged for a total score. High scores indicate high levels of psychological abuse and neglect. The CPANS has been used in some studies in Chinese adolescents (Zhang et al., 2018; Sun et al., 2019). The psychological abuse subscale was used in the present study, and its Cronbach’s alpha was 0.86.

#### Rosenberg Self-Esteem Scale

The Rosenberg Self-Esteem Scale (SES), developed by Rosenberg (1965), is a 10-item self-reported questionnaire which is used to assess self-esteem. The Chinese version of SES was developed by Ji and Yu (1999). In accomplishing the questionnaire, participants rated each item (e.g., *I think I have many good qualities*) on a 4-point scale (from 1 = *very conformity* to 4 = *very inconformity*) and ratings were averaged to form a total score. High scores indicate high levels of self-esteem. The SES has been used in some studies among Chinese adolescents (e.g., Chen et al., 2020; Yu and Liu, 2020), and the Cronbach’s alpha of this scale was 0.76 in the present study. Item parching strategy was used to improve the construct validity of SES (Landis et al., 2000). Hence, SES was broken into 3 parts, namely, self-esteem1, self-esteem2, and self-esteem3.

#### Multidimensional Scale of Perceived Social Support

The Multidimensional Scale of Perceived Social Support (MSPSS; Zimet et al., 1988) is a 12-item self-reported measure for perceived social support (SP). Participants report the extent to which they agree with each item (e.g., *my family can give me emotional help and support when I need*) using a 7-point scale (from 1 = *very strongly disagree* to 7 = *very strongly agree*). The MSPSS has shown good reliability and validity under Chinese culture context in previous studies (Chou, 2000). The Cronbach’s alpha of this scale in the present study was 0.93. Like SES, item parching strategy was used to improve the construct validity of MSPSS in the current study (Landis et al., 2000) and the questionnaire was broken into 3 parts, namely, social support1, social support2, and social support3.

#### Covariate Variables

Participants’ age, gender, number of children in the family, and family income were controlled in data analysis.

#### Data Analysis

Before the data analysis, normality, missing values, and outliers were examined, and the questionnaires with more than 15% of the data missing were excluded (Davey and Savla, 2010). Descriptive analyses and Pearson correlation analyses were done using SPSS 21. Confirmatory factor analysis (CFA) was used to test measurement models and structural equation modeling (SEM) was used to estimate the structural models. Moreover, the bootstrapping method by AMOS 22 was used to examine the mediating models.

## Results

### Descriptive Analysis

The prevalence of psychological abuse was 25.66% (107/417). Results of the independent-sample *t-*tests showed a significant difference in psychological abuse according to gender [*t*(417) = 1.79, *p* < 0.001, 95%CI = (−0.221, 0.011)]. Boys (*M* = 2.32, *SD* = 0.67) reported higher psychological abuse than girls (*M* = 2.22, *SD* = 0.52). Psychological abuse was negatively associated with self-esteem (*r* = −0.26, *p* < 0.01) and social support (*r* = −0.27, *p* < 0.01), and social support was positively associated with self-esteem (*r* = −0.28, *p* < 0.01). Descriptive statistics and correlations are presented in Table 1.

**TABLE 1:** Means, standard deviations, and correlations between study variables.

	Female (201)		Male (216)				95%CI				
Variables	*M*	*SD*	*M*	*SD*	*t*	*p*	Lowerbound	Higherbound	1	2	3
1PA	1.70	0.53	1.82	0.62	−2.08	0.04	−0.23	−0.01			
2SE	2.71	0.40	2.71	0.45	0.78	0.94	−0.08	0.09	−0.26**		
3SP	4.75	1.15	4.54	1.34	1.73	0.09	−0.03	0.45	−0.27**	0.28**	

**p < 0.01. PA, psychological abuse; SE, self-esteem; SP, social support.

### Effects of Psychological Abuse on Social Support

Results of CFA showed that each scale had acceptable construct validity (Table 2). In addition, results of the SEM indicated that psychological abuse was negatively associated with social support (β = −0.35, SE = 0.11, *p* < 0.001). The models also showed a good fit to the data (Table 2; Kline, 2011).

**TABLE 2:** Model fit indexes.

		χ^2^/*df*	SRMR	RMSEA	GFI	NFI	RFI	TLI	CFI
CFA	PA	4.179	0.063	0.087	0.890	0.817	0.783	0.826	0.853
	SE	1.706	0.048	0.032	0.986	0.979	0.931	0.912	0.991
	SP	2.710	0.036	0.064	0.994	0.995	0.945	0.964	0.997
Direct model	PA-SP	3.055	0.041	0.020	0.981	0.977	0.957	0.971	0.984
Mediation model	PA-SE-SP	2.265	0.036	0.055	0.972	0.961	0.941	0.966	0.977

*PA, psychological abuse; SE, self-esteem; SP, social support.*

### Mediation Analysis

**FIGURE 1:**
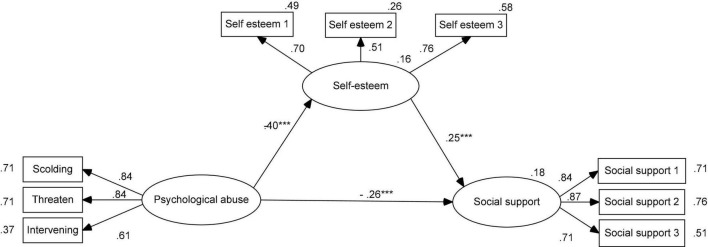
Standardized parameter estimates of the structural model demonstrating effects of psychological abuse on social support *via* self-esteem. *N* = 417. ***p < 0.001.

Based on Wen et al. (2005), two models should be estimated for mediation analysis, including direct models and mediation models. Self-esteem was a mediator that was placed into the direct model. The SEM results showed that the mediation model provided a good fit to the data (Table 2; Kline, 2011) and indicate the relationships between variables. SEM results are presented in Figure 1.

The bootstrapping method was used to estimate the mediating effects. In this mediation model (Figure 1), psychological abuse was negatively associated with self-esteem (β = −0.40, *p* < 0.001) and social support (β = −0.26, *p* < 0.001), while self-esteem was positively associated with social support (β = 0.25, *p* < 0.001). Results of a bias-corrected percentile method showed that the 95% confidence intervals (CI) of indirect effects was [−0.322, −0.089] and that the 95% CI of direct effect was [−0.742, −0.225]. Self-esteem partially mediated the relationships between psychological abuse and social support in Chinese adolescents. In this mediation model, the rate of total mediation effect to total effect was 28.57% (0.10/0.35).

## Discussion

The present study delineated the prevalence of psychological abuse and explored the pathways from psychological abuse to social support under the Chinese culture, which may broaden the scopes of psychological abuse in Chinese samples. Results showed that the prevalence of psychological abuse was 25.66%, and boys got significantly higher scores on psychological abuse than girls. Moreover, psychological abuse was negatively associated with social support and self-esteem. Furthermore, self-esteem partially mediated the relationships between psychological abuse and social support in Chinese adolescents.

Results showed that the prevalence of psychological abuse was higher than in high-income countries (Gilbert et al., 2009). This was in line with our hypothesis. In addition, the prevalence of psychological abuse in the current study was lower than in other Chinese studies (Chen, 2016; Sun et al., 2017; Cheng et al., 2018). There are several possible reasons for this inconsistency. Firstly, different cultures may contribute to different prevalence of psychological abuse. Chinese people may be more conservative than Western people, therefore causing them to have difficulties in communicating with their children. Secondly, family socioeconomic status (SES; e.g., parents’ education, occupations, and income) may influence the parenting strategies which may contribute to different prevalence of psychological abuse within Chinese samples (Liao et al., 2011). Thirdly, the economic development of China varies per area, which may be a factor in participants’ aspects of daily life. Consequently, it may also contribute to the difference in prevalence observed in this study compared to other studies conducted in other provinces. For example, the participants of the current study were recruited from Jiangsu province, a well-developed province with a different subculture from less developed provinces.

Moreover, the results showed that male adolescents were more likely to get high scores on psychological abuse than female adolescents, which is in line with our hypothesis and with some previous studies (Wang et al., 2012; Feng et al., 2015). Inconsistency with other studies (Unite States Department of Health and Human Services, 2016) may be due to the difference in cultures. Chinese traditional culture emphasizes different social roles between boys and girls, which may contribute to different parenting behaviors of parents based on gender. It also constructs much stricter rules for emotional expression and interactions among men, possibly influencing the current parenting strategies for boys. Similarly, men may have more delinquent behaviors than women (Tapper and Boulton, 2004; Schober et al., 2009), which may cause parents to more often conduct discipline on boys.

These findings suggest that psychological abuse may be an important issue among Chinese adolescents. Therefore, governments and communities should provide some information about positive parenting. For example, free reports about positive parenting strategies (e.g., parent-child commutation skills) for parents may be provided to decrease the prevalence of psychological abuse.

Meanwhile, the results showed that, consistent with previous studies, psychological abuse was negatively associated with self-esteem and social support (Jiang et al., 2010; Ma et al., 2011; Brodski and Hutz, 2012; Tian, 2014; Badr et al., 2018). According to the Attachment Theory, individuals who were exposed to psychological abuse may have biased internal work models about interpersonal relationships, contributing to their disorganized evaluations for self and self-esteem (González-Díez et al., 2016; Malik and Kaiser, 2016). Also, the individual who was exposed to psychological abuse may lack the ability to communicate with others, causing withdrawal and avoidance (Banducci et al., 2016).

Furthermore, results showed that self-esteem plays a partially mediating role in the relationships between psychological abuse and social support, which is in line with our hypothesis. Individuals exposed to psychological abuse may only have a few interpersonal skills, which may cause a lack of support from others (Pepin and Banyard, 2006; Lamis et al., 2014; Jin and Wang, 2017). In addition, the influence of psychological abuse on social support was mediated by self-esteem. Therefore, psychological abuse could indirectly influence social support through self-esteem. In other words, survivors of psychological abuse who have high levels of self-esteem may partially break the pathway from psychological abuse to low levels of social support.

These findings suggest that the levels of self-esteem may change the pathway from psychological abuse to social support. Governments and communities should provide some strategies for survivors of psychological abuse to overcome the influence of psychological abuse. For example, schoolteachers should pay much more attention to survivors of psychological abuse and give them positive feedbacks to improve their self-evaluations and self-esteem.

## Conclusion

Several limitations should be acknowledged in this study. Firstly, there are several types of self-esteem. The present study only discussed explicit self-esteem and therefore cannot give a whole view of the relationships between psychological abuse and self-esteem. Future studies need to verify the relationships between psychological abuse and other types of self-esteem. Secondly, adolescents may be psychologically immature or overconfident about their self-evaluation and esteem (e.g., Icenogle et al., 2019), which may cause a bias in the results. In addition, bias is a great factor in responses due to the use of self-reported scales in measuring all variables. Future research should collect data from different sources (e.g., peers, parents, and teachers) and use different methods (e.g., experiments) to ensure the accuracy of the information. Thirdly, a cross-sectional design may not give convincing evidence for the relationships between psychological abuse, social support, and self-esteem. Therefore, future studies need to use longitudinal design studies which can give the clear relationships of psychological abuse, self-esteem, and social support.

The present study delineated the prevalence of psychological abuse and explored the pathways from psychological abuse to social support under the Chinese culture context. Results showed that the prevalence of psychological abuse was 25.66% and that boys got significantly higher scores on psychological abuse than girls. Moreover, psychological abuse was negatively associated with social support and self-esteem. Self-esteem also partially mediated the relationships between psychological abuse and social support. These findings underscore the importance of increasing levels of self-esteem in interventions designed to improve that of psychologically abused adolescents’.

## Data Availability Statement

The original contributions presented in the study are included in the article/Supplementary Material, further inquiries can be directed to the corresponding author/s.

## Ethics Statement

The studies involving human participants were reviewed and approved by the Zhejiang Normal University. Written informed consent to participate in this study was provided by the participants’ legal guardian/next of kin.

## Author Contributions

CC designed the study, wrote and revised the manuscript, and guided the data analysis. SJ collected the data and did part of the data analysis, and wrote part of the manuscript. JJ collected the data and did part of the data analysis. All authors contributed to the article and approved the submitted version.

## Conflict of Interest

The authors declare that the research was conducted in the absence of any commercial or financial relationships that could be construed as a potential conflict of interest.

## Publisher’s Note

All claims expressed in this article are solely those of the authors and do not necessarily represent those of their affiliated organizations, or those of the publisher, the editors and the reviewers. Any product that may be evaluated in this article, or claim that may be made by its manufacturer, is not guaranteed or endorsed by the publisher.
